# The effects of nutrition bio-shield superfood powder on immune system function: A clinical trial study among patients with COVID-19

**DOI:** 10.3389/fimmu.2022.919402

**Published:** 2022-08-25

**Authors:** Farid Azizi Jalilian, Gheisar Keshavarz, Salman Khazaei, Manije Nezamdoost, Seyed Hamid Hashemi, Mojgan Mamani, Nastaran Ansari, Razieh Amini, Aref Khalkhali, Arghavan Keshavarz, Erfan Ayubi, Maryam Fazeli, Rashid Heidari Moghadam, Saeid Alizadeh, Behzad Pourhossein, Ali Teimouri, Fariba Keramat, Sajad Karampour, Mohammadreza Khakzad

**Affiliations:** ^1^ Research Center for Molecular Medicine, Hamadan University of Medical Sciences, Hamadan, Iran; ^2^ Department of Medical Virology, Faculty of Medicine, Hamadan University of Medical Sciences, Hamadan, Iran; ^3^ Department of Internal Medicine, Ilam University of Medical Sciences, Ilam, Iran; ^4^ Research Center for Health Sciences, Hamadan University of Medical Sciences, Hamadan, Iran; ^5^ Department of Infectious Disease, Farabi Hospital, Iranian Social Security Organization, Mashhad, Iran; ^6^ Department of Infectious Disease, Faculty of Medicine, Hamadan University of Medical Sciences, Hamadan, Iran; ^7^ NBS Organic Company, Istanbul, Turkey; ^8^ Faculty of Pharmacy, Shahid Beheshti University of Medical Sciences, Tehran, Iran; ^9^ Social Determinants of Health Research Center, Hamadan University of Medical Sciences, Hamadan, Iran; ^10^ Radiology Department of Sina Hospital, Hamadan, Iran; ^11^ Department of Virology, Faculty of Medicine, Iran University of Medical Sciences, Tehran, Iran; ^12^ Department of Immunology, Mashhad Branch, Islamic Azad University, Mashhad, Iran

**Keywords:** nutrition bio-safety powder, immunity system function, COVID-19, ELISA - enzyme-linked immunosorbent assay, Iran

## Abstract

The present study aimed to evaluate the effects of Nutrition Bio-shield Superfood (NBS) powder on the immune system function and clinical manifestations in patients with COVID-19. We compare the effects of NBS powder on the immune system function and clinical manifestations among two different groups: 1) intervention group receiving standard treatment scheduled according to treatment guidelines plus NBS powder, and 2) control group receiving only the same standard treatment. The serum levels of IL-2, IL-6, IL-17, IFNγ, and TNFα were determined after four weeks of treatment by specific ELISA kits according to the manufacturer’s instructions. Finally, the level of immune system stimulation and inflammatory markers were compared at baseline and after intervention in both groups. Data were analyzed using SPSS (version 22). A p-value of ≤ 0.05 was set as significant. A total of 47 patients with COVID-19 (24 patients in the intervention group and 23 patients in the control group) were included in this study. Results showed that the differences in the mean decrease of IL-2, IL-6, and TNF-α in the intervention group in comparison to the control group were 0.93, 10.28, and 8.11 pg/ml, respectively (P<0.001). On the other hand, there was no difference in IL-17, IFNγ, monocytes, eosinophil, and other inflammatory indices between the intervention and control groups. Although NBS powder was able to significantly decrease the levels of some proinflammatory cytokines in patients with COVID-19, however, it is noteworthy that the course of the disease was to large part unaffected by NBS power and there was a reduction independent of treatment. The present study indicates that NBS powder could provide a beneficial anti-inflammatory effect in patients with COVID-19. Hence, NBS in treating patients with COVID-19 shows promise as an adjuvant to the current standard antiviral treatment of such patients.

**Clinical Trial Registration:**

https://www.irct.ir, identifier IRCT20200426047206N1.

## Introduction

The ongoing pandemic of coronavirus disease 2019 (COVID-19) is considered a public health concern by World Health Organization (WHO) (1, 2). Globally, COVID-19 affects different people and is associated with significant mortality and morbidity. Till 7/July/2022 around 550,218,992 cases of COVID-19 and 6,343,783 deaths have been reported, worldwide. (https://www.worldometers.info/coronavirus/). Moreover, this new virus led to more than 7,244,694 confirmed cases and 141,420 deaths up to 8 July 2022 in Iran. (https://www.worldometers.info/coronavirus/country/iran/) (3). In general, host immune systems interact with microbial and non-microbial antigens and may stimulate cytokine production (4, 5). It was found that the immune response may play a vital role in the infectious disease caused by COVID-19. The secretion of inflammatory cytokines and other mediators of the immune system plays an important role against viral infections (6, 7). Pathogenic mechanisms of COVID-19 on the host immune systems are through decreasing the counts of lymphocytes, especially CD4+ T cell, CD8+ T cell, and B cell, and inducing excessive inflammatory reaction known as the cytokine storm phenomenon (8–10). An extremely robust cytokine storm due to an unsteady response can be extremely damaging to lung tissue and leads to lung capacity reduction, more severe disease, and even death (11). The increased level of some cytokines, including interleukin (IL)-1β, IL-2, IL-6, IL-7, IL-8, IL-10, granulocyte-colony stimulating factor (G-CSF), granulocyte macrophage-colony stimulating factor (GM-CSF), interferon-inducible protein-10 (IP10), monocyte chemotactic protein 1 (MCP1), macrophage inflammation protein-1α, IFN-γ, and tumor necrosis factor-alpha (TNF-α) is known as the signs of cytokines storm caused by COVID-19 (12, 13). It was highlighted that COVID-19 could consequently lead to a weak production of type I interferons (IFN-Is) (14). Designing and conducting clinical trials to develop new laboratory technics and drugs, to detect and treat COVID-19 is an urgent need. Nutrition Bio-Shield (Agri Bio Nutrition^®^, Turkey) (NBS) powder is an herbal product mainly made from wheat germ (15). Wheat germ contains α-linolenic acid; glutathione; fibers; minerals; tocopherols; carotenoids; B group vitamins; phytosterols; policosanols; betaine; alkylresorcinol; and polyphenols such as flavonoids, lignans, and ferulic acid (16). This herb can be used with no side effects and it has an approved number as a dietary supplement (006633-14.11.2019). Moreover, it was issued by the Turkish ministry of agriculture and forestry. Herbal medicines are natural, so they appear to cause fewer side effects (15, 17). NBS has various health benefits and may be used as a good therapeutic target in patients with COVID-19. Administration of this viable herbal supplement could improve the functioning of the immune system (15). In general, many efforts are being made in all over the world to find appropriate herbal medicine for the treatment of COVID-19 (6). However, further clinical trials are needed to better understanding about the inflammatory responses associated with COVID-19 when applying newer treatment interventions. Therefore, the present randomized controlled trial study was designed with the aim of evaluating the effect of NBS powder on immune system markers in patients with COVID-19.

## Materials and methods

### Study design

The current study was a double-blinded randomized controlled trial (RCT) study performed in Sina hospital, Hamadan, Iran from May to July 2020. In the first step, using simple randomization, all patients included were randomly assigned to one of two groups in an allocation ratio of 1:1, as follows: intervention group receiving standard treatment scheduled according to treatment guidelines plus NBS powder, and 2) control group receiving only the same standard treatment. Written informed consent was obtained from all patients. A randomized list was generated by an online randomization site. All included patients and researchers were blinded to the allocation assignments; however, the physician and clinicians team were aware of the group that was given the NBS powder.

### Participants

A total of 47 patients with COVID-19 were included in the present study. All patients were divided into two groups, the intervention group (n=24) and the control group (n=23) for four weeks. The inclusion criteria were the confirmed COVID-19 patients through PCR over the age of 20 years and who are not allergic to the powder used. Moreover, exclusion criteria were disagreement of the patient or relatives to participate in the project.

### Intervention

Patients in the intervention group received NBS powder in addition to the standard antiviral treatment. The dosage of NBS was 500 mg capsules daily in four capsules (two grams) given in divided doses of one gram in the morning and one gram in the evening for 4 weeks. Patients in control groups received standard antiviral treatment only. Standard antiviral treatment includes a two-drug regimen: Hydroxychloroquine (Oxiklorin, Elyson Pharmaceutical Co. Ltd., Seoul, Korea) and Kaletra (Lopinavir + Ritonavir) (AbbVie Inc. North Chicago, Illinois, U.S.A).

### ELISA assay

For this purpose, at the end of the fourth week, blood samples were taken from each patient. We evaluated the serum levels of IL-2, IL-6, IL-17, IFNγ, and TNFα with ELISA kits (ZellBio Co., Germany) according to the manufacturer’s instructions. Results were analyzed by an ELISA microplate reader (Bio Tek E1800, USA), and the concentrations were calculated.

### Statistical methods

Data were analyzed using SPSS (version 22). A *P*-value of ≤ 0.05 was set as significant. Shapiro-Wilk test was used to check whether parameters were normally distributed. Parameters with normal and non-normal distribution were presented using mean (standard deviation; SD) and median (interquartile range; IQR) according to treatment groups, respectively. Paired t-test and Wilcoxon signed-rank test were used for comparison baseline vs. end line within each treatment group, as appropriate. Comparison between the treatment groups was carried out by independent t-test and Mann-Whitney test, as appropriate (6, 18, 19).

## Results

**Figure 1:**
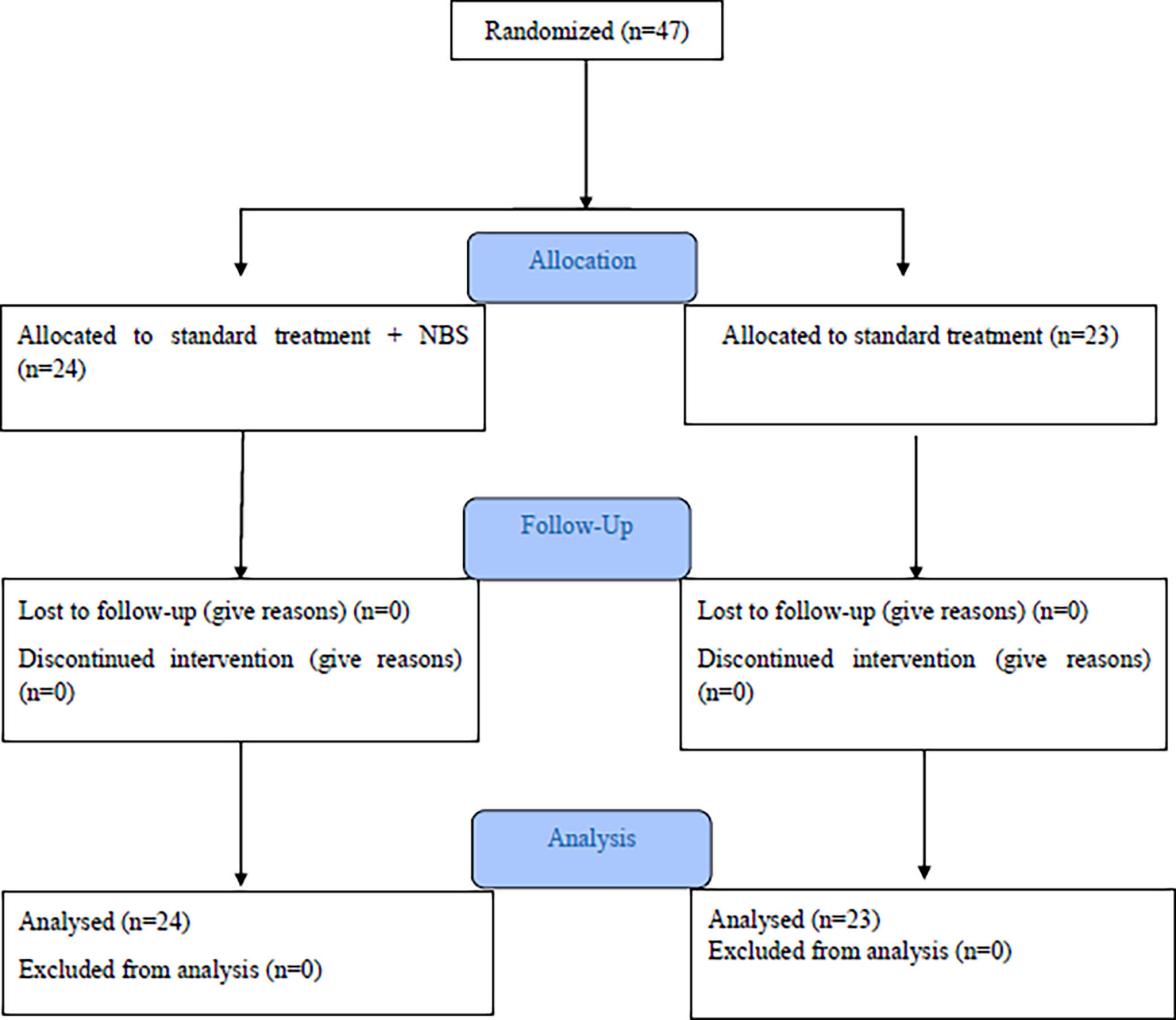
Consort flow chart.

Among the participants, 24 patients received NBS powder in addition to the standard antiviral treatment (intervention group) randomly, and 23 patients received standard treatment as the control group (**Figure 1**). Mean ± SD age of all patients in intervention and control groups were 51.54 ± 16.66 years (range; 22 to 87 years) and 46.26 ± 18.69 (range; 22 to 82 years), respectively. **Table 1** illustrates the characteristics of the patients. There were no significant differences in all characteristics of patients between the two treatment groups. Age-sex distribution of patients was similar between the two groups. The frequency of smoking among patients in the control group was higher than those in another group (17.39% vs. 12.50%). Approximately 70% of the patients had underlying diseases in the two groups.

**Table 1:** Characteristics of patients in intervention and control groups.

	Standard treatment plus NBS powder (n = 24)	Standard treatment (n = 23)	P-value
**Age (year)**			
**<60**	18 (78.26%)	17 (77.27%)	0.94
**≥60**	5 (21.74%)	5 (22.73%)	
**Gender**			
Male	7 (29.17%)	7 (30.43%)	0.92
Female	17 (70.83%)	16 (69.57%)	
**Education**			
Illiterate	6 (25%)	5 (21.74%)	0.19
Primary	9 (37.50%)	13 (56.52%)	
High school	5 (20.83%)	5 (21.74%)	
Academic	4 (16.67%)	0 (0%)	
**Residence**			
Urban	19 (79.17%)	17 (73.91%)	0.67
Rural	5 (20.83%)	6 (26.09%)	
**Marital status**			
Single	3 (12.50%)	5 (21.74%)	0.40
Married	21 (87.50%)	18 (78.26%)	
**Occupation**			
Unemployed	15 (65.22%)	16 (69.57%)	0.75
Employed	8 (34.78%)	7 (30.43%)	
**Smoking**			
Yes	3 (12.50%)	4 (17.39%)	0.64
No	21 (87.50%)	19 (82.61%)	
**Underlying disease**			
Yes	17 (70.83%)	16 (69.57%)	0.92
No	7 (29.17%)	7 (30.43%)	

**Figure 2:**
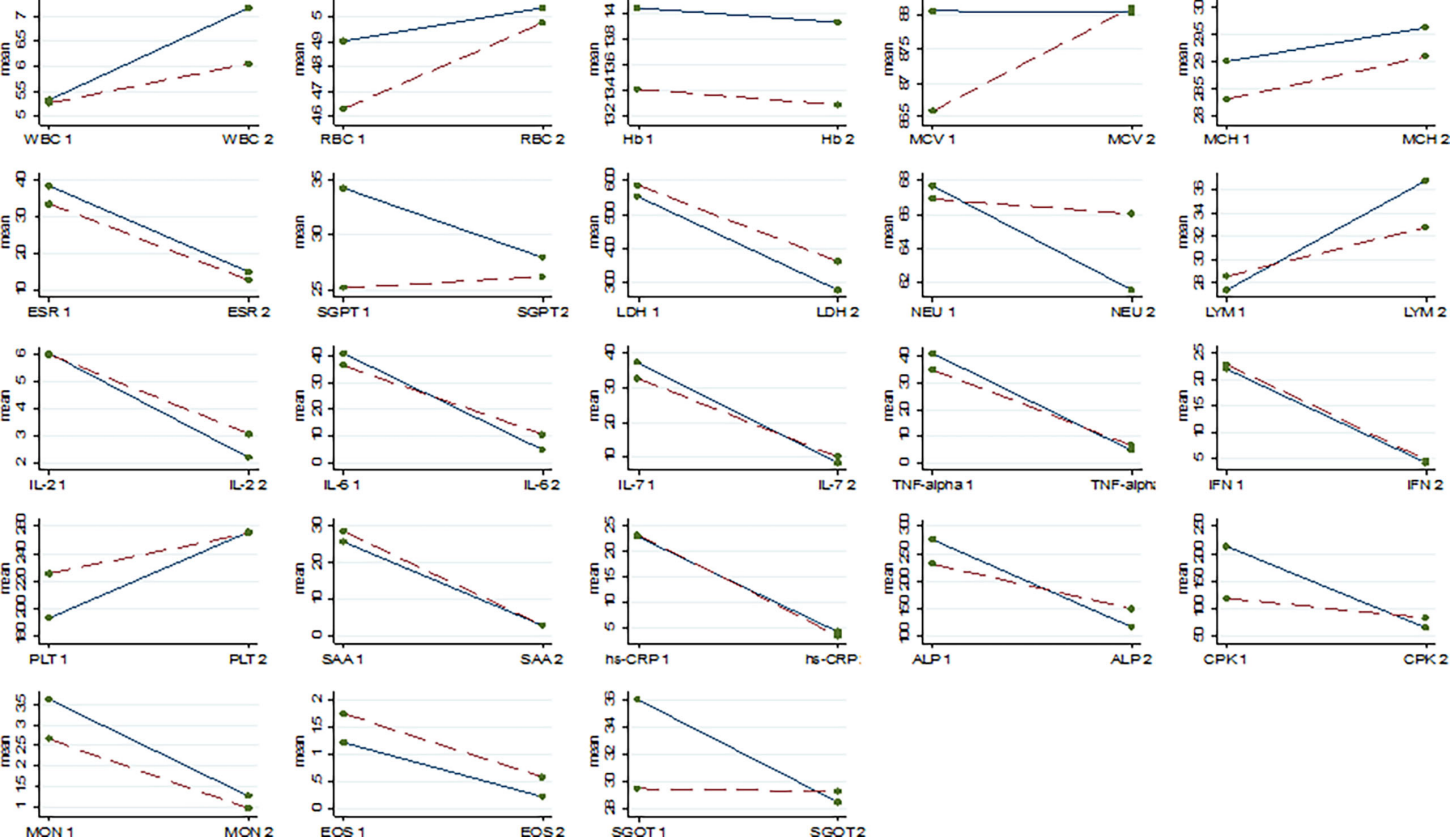
The change of mean of laboratory parameters from baseline (1) to end line (2); White Blood Cell (WBC), Red Blood Cell (RBC), Hemoglobin (HB), Mean Corpuscular Volume (MCV), Mean Corpuscular Hemoglobin (MCH), Erythrocyte Sedimentation Rate (ESR), Serum Glutamate-Pyruvate Transaminase (SGPT), Lactate Dehydrogenase (LDH), Neutrophil (NEU), Lymphocyte (LYM), Interleukin-2 (IL-2), Interleukin 6 (IL-6), Interleukin 7 (IL-7), Tumor Necrosis Factor (TNF)-alpha, Interferon (IFN), Platelet (PLT), Serum Amyloid A (SAA), High-Sensitivity C-Reactive Protein (hs-CRP), Serum Glutamic-Oxaloacetic Transaminase (SGOT), Alkaline Phosphatase (ALP), Creatine Phosphokinase (CPK), Monocytes (MON), Eosinophil (EOS); the change for Standard + NBS treatment group is solid line, the change for Standard treatment only group is Dash line.

Results of comparing the level of parameters in the baseline and after the intervention within the groups were presented in **Table 2** and **Figure 2**. A significant difference was found between the baseline and after the intervention in all of the studied parameters (P<0.05) except for the hemoglobin test (Hb), mean corpuscular volume (MCV), and mean corpuscular hemoglobin (MCH). **Table 3** illustrated the comparing of difference in the level of parameters between the two groups at the end of follow-up. Statistical analysis showed a greater mean reduction in the level of cytokines including IL-2, IL-6, and TNF-α in the intervention group than in the control group (P<0.001), e.g. ^Δ^ mean difference for IL-2, IL-6, IL-17, and TNF-α were 0.93, 10.28, and 8.11, respectively. Although NBS powder was able to significantly decrease the levels of some proinflammatory cytokines in patients with COVID-19, however, it is noteworthy that the course of the disease was to large part unaffected by NBS power compared to the standard treatment. In addition, the level of SGPT, SGOT, NEU, ALP, and CPK tends to decrease more rapidly in the patients in the intervention group than in those in the control group (P<0.05). The statistical analysis showed that the level of white blood cells (WBC) and lymphocytes (LYM) in the intervention group increased significantly more than in the control group e.g. Δ mean difference for WBC and LYM was 1.05 and 5.12, respectively.

**Table 2:** Level of parameters in baseline and endline within intervention and control groups.

	Standard treatment plus NBS powder (n = 24)			Standard treatment (n = 23)		
Normally distributed parameters	Baseline	Endline	P*	Baseline	Endline	P*
WBC (×10^9^/L)	5.33 (1.68)	7.16 (1.44)	<0.001	5.27 (1.70)	6.05 (1.45)	<0.001
RBC (×10^6^/µL)	4.90 (0.46)	5.04 (0.58)	0.26	4.63 (0.88)	4.97 (0.61)	0.02
Hb (g/dl)	14.04 (1.61)	13.93 (1.45)	0.17	13.41 (1.63)	13.29 (1.50)	0.20
MCV (fL)	88.06 (6.42)	88.04 (3.34)	0.98	86.58 (6.56)	88.11 (3.88)	0.09
MCH (pg)	29.02 (2.77)	29.64 (1.62)	0.11	28.32 (3.05)	29.11 (1.63)	0.06
ESR (mm/hr)	38.5 (22.82)	14.79 (7.33)	<0.001	33.65 (19.72)	12.56 (6.85)	<0.001
SGPT (IU/L)	34.29 (18.80)	27.95 (10.48)	0.01	25.17 (10.98)	26.17 (8.98)	0.20
LDH (U/L)	554.41 (183.44)	281.41 (40.68)	<0.001	586.04 (143.08)	364.78 (60.09)	<0.001
NEU (×10^3^/mm^3^)	67.67 (10.13)	61.58 (4.61)	0.003	66.91 (10.77)	66.04 (3.56)	0.63
LYM (×10^3^/mm^3^)	27.46 (9.06)	36.75 (3.86)	<0.001	28.61 (9.38)	32.78 (4.23)	0.03
IL-2 (pg/mL)	6.03 (1.82)	2.19 (0.79)	<0.001	5.99 (1.37)	3.06 (0.78)	<0.001
IL-6 (pg/mL)	41.04 (17.06)	4.85 (2.45)	<0.001	36.48 (13.12)	10.57 (4.07)	<0.001
IL-17 (pg/mL)	37.33 (14.40)	8.37 (3.65)	<0.001	32.74 (15.16)	10.03 (4.01)	<0.001
TNF-α (pg/mL)	41.21 (13.08)	4.40 (1.46)	<0.001	35.08 (7.78)	6.39 (1.78)	<0.001
IFN-γ (pg/mL)	22 (3.77)	4.36 (1.02)	<0.001	22.95 (4.56)	4.83 (1.06)	<0.001
Non-normally distributed parameters	**Baseline**	**Endline**	**P**¶	**Baseline**	**Endline**	**P**¶
PLT (×10^3^/mm^3^)	188 (79.5)	245 (79.5)	<0.001	207 (114)	241 (52)	0.02
SAA (U/mL)	12 (31.5)	2 (1)	<0.001	12 (16)	2 (1)	<0.001
hs-CRP (mg/L)	19.5 (12.5)	4 (2)	<0.001	20 (24)	3 (1)	<0.001
SGOT (IU/L)	31.5 (19.5)	30 (13.5)	0.002	26 (8)	27 (6)	0.53
ALP (IU/L)	211 (128.5)	102.5 (48.5)	<0.001	229 (138)	150 (64)	<0.001
CPK (U/L)	114 (102.5)	55 (16)	0.001	108 (73)	87 (25)	<0.001
MON (×10^3^/mm^3^)	3 (1.5)	1 (2)	<0.001	2 (2)	1 (2)	<0.001
EOS (×10^3^/mm^3^)	1 (2)	0 (0)	0.003	2 (3)	0 (1)	<0.001

WBC, White Blood Cell; RBC, Red Blood Cell; HB, Hemoglobin; MCV, Mean Corpuscular Volume; MCH, Mean Corpuscular Hemoglobin; ESR, Erythrocyte Sedimentation Rate; SGPT, Serum Glutamate-Pyruvate Transaminase; Lactate Dehydrogenase van der Wal, Jaarsma, & van Veldhuisen), Neuman et al., neutrophil; LYM, Lymphocyte; IL-2, Interleukin-2; IL-6, Interleukin 6; IL-17, Interleukin 17 TNF-alpha, Tumor necrosis factor; (& AshayeriNori, Seifnaraghi) Interferon; PLT, platelet; serum amyloid A (Kasliwal, Wilton, Cornelius, Aurich-Barrera, & Shakir), hs-CRP, High-sensitivity C-reactive protein; (SGOT, Serum Glutamic-Oxaloacetic Transaminase; ALP, Alkaline Phosphatase; CPK, Creatine Phosphokinase; MON, Monocytes; EOS, Eosinophil.

Normally and non-normally distributed parameters were presented as mean (SD) and median (IQR), respectively.

* paired t-test, ¶ Wilcoxon signed-rank test.

**Table 3:** Comparing of difference in level of parameters between the intervention and control groups at the end of follow up.

	Standard treatment plus NBS powder (n = 24)	Standard treatment (n = 23)	
Normally distributed parameters	Difference	Difference	P**
WBC (×10^9^/L)	1.83 (1.28)	0.78 (0.69)	**0.001**
RBC (×10^6^/µL)	0.13 (0.57)	0.34 (0.69)	0.26
Hb (g/dl)	-0.11 (0.39)	-0.12 (0.44)	0.94
MCV (fL)	-0.02 (4.50)	1.53 (4.21)	0.23
MCH (pg)	0.62 (1.84)	0.79 (1.94)	0.77
ESR (mm/hr)	-23.71 (16.83)	-21.08 (13.99)	0.56
SGPT (IU/L)	-6.33 (11.51)	1 (3.63)	**0.005**
LDH (U/L)	-273 (175.29)	-221.26 (118.20)	0.24
NEU (×10^3^/mm^3^)	-6.08 (9.09)	-0.87 (8.67)	**0.05**
LYM (×10^3^/mm^3^)	9.29 (8.60)	4.17 (8.65)	**0.05**
IL-2 (pg/mL)	-3.84 (1.91)	-2.91 (0.92)	**0.04**
IL-6 (pg/mL)	-36.19 (15.37)	-25.91 (11.51)	**0.01**
IL-17 (pg/mL)	-28.96 (12.22)	-22.70 (12.19)	0.08
TNF-α (pg/mL)	-36.80 (12.35)	-28.69 (6.97)	**0.008**
IFN-γ (pg/mL)	-18.64 (3.65)	-18.12 (4.15)	0.67
Non-normally distributed parameters	**Difference**	**Difference**	**P**§
PLT (×10^3^/mm^3^)	64 (94.5)	57 (98)	0.28
SAA (U/mL)	-10 (31.5)	-9 (15)	0.61
hs-CRP (mg/L)	-16.5 (11.5)	-18 (24)	0.76
SGOT (IU/L)	-2.5 (7)	1 (3)	**0.005**
ALP (IU/L)	-112 (141)	-83 (93)	**0.04**
CPK (U/L)	-58.5 (104.5)	-23 (48)	**0.03**
MON (×10^3^/mm^3^)	-2 (2)	-2 (2)	0.32
EOS (×10^3^/mm^3^)	0 (2)	-1 (2)	0.42

WBC, White Blood Cell; RBC, Red Blood Cell; HB, Hemoglobin; MCV, Mean Corpuscular Volume; MCH, Mean Corpuscular Hemoglobin; ESR, Erythrocyte Sedimentation Rate; SGPT, Serum Glutamate-Pyruvate Transaminase; van der Wal et al., Lactate Dehydrogenase; Neuman et al., Neutrophil; LYM, Lymphocyte; IL-2, Interleukin-2; IL-6, Interleukin 6; IL-17, Interleukin 17; TNF-alpha, Tumor Necrosis Factor; Nori et al., Interferon; PLT, Platelet; Kasliwal et al., Serum Amyloid A; hs-CRP, High-Sensitivity C-reactive Protein; SGOT, Serum Glutamic-Oxaloacetic Transaminase; ALP, Alkaline Phosphatase; CPK, Creatine Phosphokinase; MON, Monocytes; EOS, Eosinophil.

Normally and non-normally distributed parameters were presented as mean (SD) and median (IQR), respectively.

** Independent t-test, § Mann-Whitney test. Bold values: P value ≥ 0.05.

## Discussion

Multiple organ dysfunction syndrome, acute cardiac complications, acute respiratory distress syndrome, septic shock, and death are the severe complications of COVID-19 infection (20, 21). It is revealed that these manifestations and complications are related to viral replication that induces an abnormally strong release of cytokines and an excessive inflammatory reaction known as the cytokine storm (22). Several previously published studies revealed that optimal dietary patterns and adequate nutrient status are significant to modulate inflammatory reaction and oxidative stress processes. Moreover, in most cases, an optimal immune response is related to optimal dietary and nutritional constituents and is fundamental to preventing infection (23–25). In general, different plant-based foods can exert anti-inflammatory and antioxidant properties (26, 27). In the present RCT study, we aimed to evaluate the effects of NBS powder on the immune system function and clinical manifestations in patients with COVID-19. As far as we are concerned, the current study is the first research to have evaluated the effect of NBS powder, on immune system function in patients with COVID-19. In the present study we found a difference in the reduction of levels of IL-2, IL-6, and TNF-α. Results of our study showed that inflammatory responses in the patients who received NBS powder for four weeks with standard treatment (intervention group) were induced statistically fewer than in the patients who received only the standard treatment. Our finding revealed that compared to the control group, the means of IL-2, IL-6, and TNF-α concentrations were reduced by 0.93, 10.28, and 8.11 per unit during 4 weeks follow-up when NBS powder was added to the standard treatment in the intervention group.

Likewise, a similar pattern was found for both IL-17 and IFN-γ; however, the result was not statistically significant. Results of the current study revealed that NBS powder significantly decreased the levels of some proinflammatory cytokines in patients with COVID-19, however, it is noteworthy that the course of the disease was to large part unaffected by NBS power compared to the standard treatment. Therefore, there was a reduction independent of treatment.

The observed change in other inflammatory and immunity indices supports the clinical benefits of adding NBS powder to the standard treatment. The evidence also indicated that the severity of COVID-19 increases with the levels of inflammatory mediators including cytokines and chemokines such as IL-2, IL-7, IL-10, and TNF-α in the blood due to COVID-19 infection (28, 29). Significantly, by comparing survivors and non-survivors of COVID-19, it was observed that IL-6 plays a more important role in mortality than the other increased inflammatory factors (30). No statistical significance of ^Δ^ mean difference for IL-17 between the two groups may be due to the small sample size of the study that reduced the power of the study, thus there is a possibility of type II error in the results of this study. In the previously published RCT study (31), the researchers found that patients with COVID-19 receiving lopinavir-ritonavir along with interferon beta-1b and ribavirin had a significantly lower level of IL-6 at days 2, 6, and 8 in comparison with patients who only received lopinavir and ritonavir. Moreover, their results for TNF-α and IL-10 concentrations were not significant. To date, several studies have been performed on the effects of wheat germ (as the main component of NBS) and its derivatives on immune responses. Hussein et al. (2014) reported that pretreatment with wheat germ oil in a rat model of endotoxemia led to the suppression of serum levels of TNF-α and IL-6 (32). This finding is consistent with the results obtained in our study. In another study (2019) by Ojo et al., it was shown that wheat germ could reduce serum concentrations of IL-1b, IL-6, INF-γ, and TNF-α in C57BL6 mice. This study suggested that the addition of this nutrient to the diet may be vital in preventing diet-induced comorbidities (33). The present study was a double-blinded, randomized, controlled clinical trial, which could be considered a prerequisite to detect a treatment effect of an intervention. In addition, the distribution of the patients’ characteristics at baseline was balanced across the two groups, which decreased the chance of confounding in the results of the study. It was observed that the prevention of inflammatory responses to cytokines or their dependent receptors by antibodies or neutralizing compounds can decrease immune-mediated damage (34). The obtained data from the treatment with NBS in the patients of the test group showed a decrease in concentration of inflammatory cytokine. We showed that NBS significantly reduced the levels of IL-2, IL-6, and TNF-α; therefore, it can be considered as a potential treatment option that may reduce side effects of the COVID-19 disease.

## Conclusion

The results of the present RCT show that NBS powder combined with the standard treatment might prevent inflammatory responses against COVID-19 infection. In this study, the standard treatment, which could preclude detecting the action mechanism of NBS, was administered to both groups. Moreover, our findings are preliminary. Therefore, larger RCTs with repeated measurements and large and different intervention groups are needed to determine the beneficial effects of NBS on the immune system markers.

## Data availability statement

The original contributions presented in the study are included in the article/supplementary material. Further inquiries can be directed to the corresponding author.

## Ethics statement

The trial was approved by the Ethics committee of Hamadan University of Medical Sciences (IR.UMSHA.REC.1399.046; approval date: 2020-05-19) and conducted according to the Helsinki Declaration. The patients/participants provided their written informed consent to participate in this study.

## Author contributions

FK, FAJ, GK, SKh, and MN: Conceptualization; Data curation; Formal analysis; and Writing – original draft. SH, MM, NA, RA, AKh, and AKe: Conceptualization; Methodology; Project administration; and Writing – original draft. EA, MF, RH, SA, and BP: Data curation; Formal analysis; Writing – original draft; and Writing – review and editing. AT, FK, SKa, and MK: Language editing. All authors contributed to the article and approved the submitted version.

## Conflict of interest

The authors declare that the research was conducted in the absence of any commercial or financial relationships that could be construed as a potential conflict of interest.

## Publisher’s note

All claims expressed in this article are solely those of the authors and do not necessarily represent those of their affiliated organizations, or those of the publisher, the editors and the reviewers. Any product that may be evaluated in this article, or claim that may be made by its manufacturer, is not guaranteed or endorsed by the publisher.
